# SARS-CoV-2 Transmission from Virus-Infected Dead Hamsters

**DOI:** 10.1128/msphere.00411-22

**Published:** 2023-01-10

**Authors:** Kiyoko Iwatsuki-Horimoto, Hiroshi Ueki, Mutsumi Ito, Sayaka Nagasawa, Yuichiro Hirata, Kenichiro Hashizume, Kazuho Ushiwata, Hirotaro Iwase, Yohsuke Makino, Tetsuo Ushiku, Shinji Akitomi, Masaki Imai, Hisako Saitoh, Yoshihiro Kawaoka

**Affiliations:** a Division of Virology, Institute of Medical Science, University of Tokyo, Tokyo, Japan; b The Research Center for Global Virus Diseases, National Center for Global Health and Medicine Research Institute, Tokyo, Japan; c Department of Legal Medicine, Graduate School of Medicine, Chiba University, Chiba, Japan; d Department of Pathology, National Institute of Infectious Diseases, Tokyo, Japan; e GSI Co., Ltd., Tokyo, Japan; f Department of Forensic Medicine, Graduate School of Medicine, University of Tokyo, Tokyo, Japan; g Department of Pathology, Graduate School of Medicine, University of Tokyo, Tokyo, Japan; h Japan Medical Association Research Institute, Tokyo, Japan; i Influenza Research Institute, Department of Pathobiological Sciences, School of Veterinary Medicine, University of Wisconsin—Madison, Madison, Wisconsin, USA; j The University of Tokyo, Pandemic Preparedness, Infection, and Advanced Research Center, Tokyo, Japan; Emory University School of Medicine

**Keywords:** SARS-CoV-2, COVID-19, dead body, transmission, embalming, animal model

## Abstract

Although it has been 2.5 years since the coronavirus disease 2019 (COVID-19) pandemic began, the transmissibility of severe acute respiratory syndrome coronavirus 2 (SARS-CoV-2) from a dead infected body remains unclear, and in Japan, bereaved family members are often not allowed to view in person a loved one who has died from COVID-19. In this study, we analyzed the possibility of SARS-CoV-2 transmission from a dead body using a hamster model. We also analyzed the effect of “angel care”––in which the pharynx, nostrils, and rectum are plugged––and embalming on reducing transmissibility from dead bodies. We found that SARS-CoV-2 could be transmitted from the bodies of animals that had died within a few days of infection; however, angel care and embalming were effective in preventing transmission from the dead bodies. These results suggest that protection from infection is essential when in contact with a SARS-CoV-2-infected dead body and that sealing the cavities of a dead body is an important infection control step if embalming is not performed.

**IMPORTANCE** We found that SARS-CoV-2 could be transmitted from a dead body, presumably via postmortem gases. However, we also found that postmortem care, such as plugging the pharynx, nostrils, and rectum or embalming the corpse, could prevent transmission from the dead body. These results indicate that protection from infection is essential when handling infected corpses and that appropriate care of SARS-CoV-2-infected corpses is important.

## INTRODUCTION

In July 2020, detailed procedures for the transportation, funeral, and cremation of those who died from coronavirus disease 2019 (COVID-19) were established by the federal government in Japan (1). These guidelines offered several infection prevention and control strategies, including recommending that the family members of the deceased refrain from touching or coming into close contact with the dead body to avoid the potential for infectious risks; stating that the dead body must be contained, sealed in a nonpermeable body bag, and not opened; and recommending that cremation be performed within 24 h, although this was not mandatory. In fact, in most cases, the bereaved family members were not able to view their loved one in person, and the cremains were given to the families after cremation had taken place. In May 2022, a guide to medical care issued by the Japanese government stated, “It is allowed for the bereaved family to see the deceased face-to-face in an appropriately infection-controlled hospital room” (2). However, to this day, many medical institutions still do not allow bereaved family members to view their loved ones who have died from COVID-19.

There have been reports of infectious severe acute respiratory syndrome coronavirus 2 (SARS-CoV-2) being detected in the bodies of those who have died from COVID-19 (3–5); however, it is not clear whether the virus can be transmitted from such bodies. In Japan, the most common way for nurses in hospitals to care for dead bodies is to wipe the surface of the face, neck, hands, and feet with alcohol-soaked cotton, in addition to taking care of the appearance of the deceased by, for example, shaving them or applying cosmetics. In addition, the mouth, nose, ears, and anus are stuffed with cotton pads to prevent leakage of bodily fluids. This postmortem care of deceased individuals is known as “angel care” in Japan (6). Embalming, which is widely used in the United States and Canada, has recently been on the rise in Japan. However, there are no reports of whether angel care or embalming reduce the infectivity of SARS-CoV-2 when applied to those who have died as a result of COVID-19, and the actual infectivity of SARS-CoV-2 from an infected dead body is unknown. Accordingly, in this study, we analyzed the possibility of SARS-CoV-2 transmission from dead bodies and the effect of angel care and embalming on the transmissibility of SARS-CoV-2 from infected dead bodies using a hamster model.

## RESULTS

### Transmissibility of SARS-CoV-2 from the dead body of an infected hamster.

**FIG 1:**
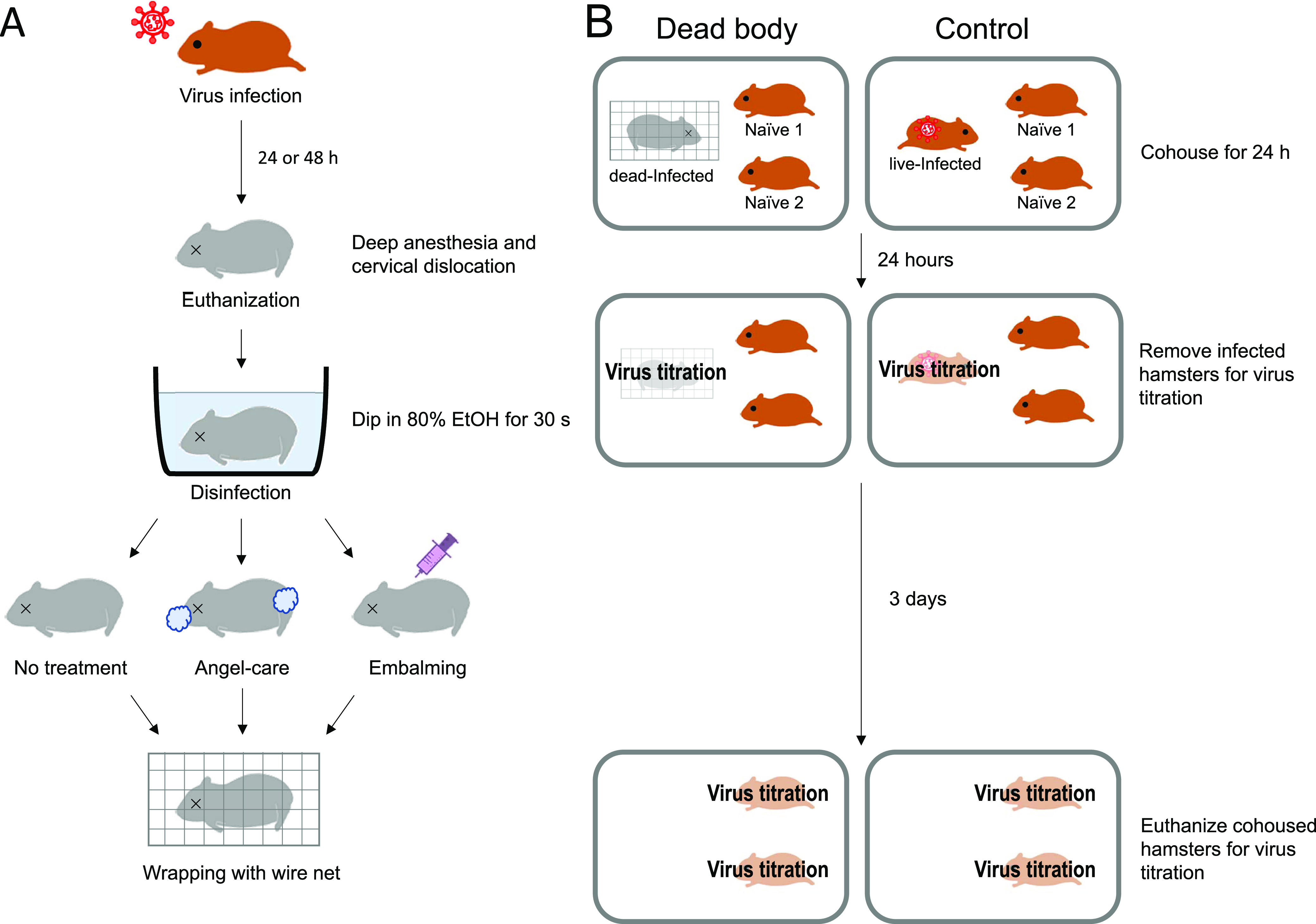
Schematic representation of the treatment of the dead bodies. (A) Syrian hamsters were euthanized at 24 or 48 h postinfection, disinfected, treated, and wrapped with wire net to prevent cannibalization. (B) One wrapped body and two naive hamsters were cohoused as one group in the same cage. As a control, one live infected hamster and two naive hamsters was also cohoused. Twenty-four hours after cohousing, the wrapped body and the live infected hamster were removed from the cages, and the organs of the dead body and the euthanized infected hamsters were collected for virus titration. The remaining naive hamsters were euthanized 3 days after removal of the infected hamster, and their organs were collected for virus titration. EtOH, ethanol.

First, we assessed the transmissibility of SARS-CoV-2 from the dead body of an infected hamster. Six-month-old Syrian hamsters infected with 10^3^ PFU of SARS-CoV-2/UT-NCGM02/human/2020 (Wuhan strain) were euthanized at 24 or 48 h postinfection with deep anesthesia and cervical dislocation. To disinfect viruses on the surface of the bodies, their entire bodies were immersed in an alcohol bath for 30 s (Fig. 1A). The bodies were then wrapped with wire net to prevent them from being cannibalized by cohousing hamsters. One wrapped body and two naive hamsters were cohoused as one group in the same cage. As a control, one live infected hamster and two naive hamsters were also cohoused (Fig. 1B). Two groups per condition were used for this study. Twenty-four hours after cohousing, the wrapped body and the live infected hamster were removed from the cages, and the organs of the dead body and the euthanized infected hamsters were collected for virus titration. The remaining naive hamsters were euthanized 3 days after removal of the infected hamster, and their organs were collected for virus titration (Fig. 1B). For the live infected hamsters, high titers of virus were found in the lungs and nasal turbinates (Table 1). SARS-CoV-2 was transmitted from all live infected hamsters under both conditions of cohousing (i.e., cohoused at 24 and 48 h postinfection). For the dead infected hamsters, at 24 h postmortem, high titers of virus remained in the lungs and nasal turbinates. Moreover, SARS-CoV-2 was transmitted from 1 of the 2 groups cohoused with a dead infected hamster under the condition of cohousing starting at 24 but not at 48 h postinfection. To confirm transmissibility from the dead body, we examined an additional 8 groups under the condition of cohousing a dead infected hamster with naive hamsters starting at 24 h postinfection. To exclude the possibility of transmission to naive hamsters by contamination from live infected hamsters, we intentionally excluded the group that was cohoused with live infected animals. Among these 8 groups, SARS-CoV-2 was transmitted from the dead body in 2 groups (Table 2). Therefore, in 3 out of 10 groups, SARS-CoV-2 was transmitted from the dead infected hamster to the naive hamsters. These results indicate that SARS-CoV-2 can be transmitted from a dead body in the early stage of infection.

**TABLE 1:** Transmissibility of SARS-CoV-2 from live animals and dead bodies^*a*^

Timing of cohousing	Condition of infected animals	Group	Virus titer (log_10_ PFU) in hamsters					
			Infected		Naive 1		Naive 2	
			Lung	NT^*b*^	Lung^*c*^	NT	Lung	NT
24 h postinfection	Live	1	8.49	9.36	8.45	8.58	8.27	8.30
		2	8.74	9.26	8.12	8.43	8.38	8.80
	Dead	1	7.72	8.42	7.32	7.26	1.84	
		2	7.74	6.42				
48 h postinfection	Live	1	8.29	8.44	5.22	7.07	7.05	8.90
		2	8.17	8.68	8.41	8.90	8.32	8.93
	Dead	1	8.15	8.43				
		2	8.04	8.69				

a Six-month-old Syrian hamsters infected with 10^3^ PFU of SARS-CoV-2 strain NCGM02 were euthanized at 24 or 48 h postinfection with deep anesthesia and cervical dislocation. One wrapped body or one live infected hamster and two SARS-CoV-2-naive hamsters were cohoused as one group in the same cage. Two groups per condition were used for this study. Twenty-four hours after cohousing, the wrapped body and the live infected hamster were removed from the cage, and the organs of the dead body and euthanized-infected hamsters were collected for virus titration. The remaining naive hamsters were euthanized three days after removal of the infected hamster, and their organs were collected for virus titration.

b NT, nasal turbinate.

c Empty cells indicate that no virus was detected (detection limit, 1.0 log_10_ PFU/mL).

**TABLE 2:** Transmissibility of SARS-CoV-2 from dead bodies^*a*^

Timing of cohousing	Condition of infected animals	Group	Virus titer (log_10_ PFU) in hamsters					
			Infected		Naive 1		Naive 2	
			Lung	NT^*b*^	Lung^*c*^	NT	Lung	NT
24 h postinfection	Dead	3	7.63	8.79				
		4	7.97	8.84				
		5	7.77	6.23				
		6	7.58	8.33				
		7	7.95	8.70	7.54	>8.0	8.18	>8.0
		8	7.75	8.47	2.50	2.61	1.90	2.16
		9	7.61	6.21				
		10	7.65	7.62				

a Six-month-old Syrian hamsters infected with 10^3^ PFU of SARS-CoV-2 strain NCGM02 were euthanized at 24 h postinfection with deep anesthesia and cervical dislocation. One wrapped body and two SARS-CoV-2-naive hamsters were cohoused as one group in the same cage. Eight groups were used for this study. Twenty-four hours after cohousing, the wrapped body was removed from the cage, and the lungs and nasal turbinates were collected for virus titration. The remaining naive hamsters were euthanized three days after removal of the body, and their collected lungs and nasal turbinates were collected for virus titration.

b NT, nasal turbinate.

c Empty cells indicate that no virus was detected (detection limit, 1.0 log_10_ PFU/mL).

### Effect of angel care on the transmission of SARS-CoV-2 from the dead body of an infected hamster.

We next examined the effectiveness of angel care in preventing transmission of SARS-CoV-2 from a dead hamster. Usually, in human angel care, the pharynx and nostrils are stuffed with moisture-absorbing gel and plugged with cotton, the rectum is stuffed with fiber and cotton, and the ears are stuffed with only cotton in order to prevent the leakage of bodily fluids. In this study, we used the same gel that is used for human angel care in the hamster’s mouth and then plugged it with cotton. Since hamsters’ nostrils and rectum are too small to perform the procedure done in humans, we used medical-grade Aron Alpha adhesive to plug these sites. No treatment was given to the ears. One wire-wrapped SARS-CoV-2-infected body that had undergone angel care and two naive hamsters were considered one group, and we examined 10 groups. To exclude the possibility of transmission to naive hamsters by contamination from live virus-infected hamsters, we intentionally excluded the group that was cohoused with live virus-infected animals. High titers of virus were still detected in the lungs and nasal turbinates of the body that was treated with angel care; however, SARS-CoV-2 was not transmitted from the body to any of the naive hamsters in any of the groups (Table 3). This result indicates that angel care was effective in preventing SARS-CoV-2 transmission from a dead body.

**TABLE 3:** Effect of angel care on virus transmission from a dead body^*a*^

Timing of cohousing	Treatment	Group	Virus titer (log_10_ PFU) in hamsters					
			Infected		Naive 1		Naive 2	
			Lung	NT^*b*^	Lung^*c*^	NT	Lung	NT
24 h postinfection	Angel care	1	7.84	7.66				
		2	7.86	8.02				
		3	7.55	8.56				
		4	7.54	8.67				
		5	7.75	7.80				
		6	7.56	7.45				
		7	2.60	6.85				
		8	7.82	8.97				
		9	7.28	6.95				
		10	7.03	7.42				

a Six-month-old Syrian hamsters infected with 10^3^ PFU of SARS-CoV-2 strain NCGM02 were euthanized at 24 h postinfection with deep anesthesia and cervical dislocation. Alcohol-dipped bodies were used for angel care. Three hundred microliters of the gel was inserted into the mouth, which was then plugged with cotton; the nostrils and rectum were plugged with medical-grade Aron Alpha adhesive. One wrapped body and two SARS-CoV-2-naive hamsters were cohoused as one group in the same cage. Eight groups were used for this study. Twenty-four hours after cohousing, the wrapped body was removed from the cage, and the organs were collected for virus titration. The remaining naive hamsters were euthanized three days after removal of the body, and their organs were collected for virus titration.

b NT, nasal turbinate.

c Empty cells indicate that no virus was detected (detection limit, 1.0 log_10_ PFU/mL).

### Effect of embalming on the transmission of SARS-CoV-2 from the dead body of an infected hamster.

Finally, we examined the effectiveness of embalming on preventing transmission. Embalming agents, the same as those used in humans, were injected through the apex of the heart, and blood was drained through the common femoral vein. The incision was sutured with a medical stapler. One wire-wrapped embalmed body and two naive hamsters were considered one group, and we examined 10 groups. The virus titer of the embalmed body could not be determined because of the toxicity of the formaldehyde to the cultured cells used for virus titration. SARS-CoV-2 was not transmitted from the embalmed body to any of the naive hamsters in any group. This result indicates that embalming was effective in preventing SARS-CoV-2 transmission from a dead body.

## DISCUSSION

In this study, we demonstrated that SARS-CoV-2 could be transmitted from a dead body to live animals in a hamster model. Subgenomic RNA, indicating viral replication of SARS-CoV-2, has been detected in specimens collected from the dead bodies of COVID-19 patients at 35.8 h postmortem (3). Another study reported that infectious viruses were isolated from the lungs of two COVID-19 corpses at 4 and 17 days postmortem, respectively (4). In yet another study, it was reported that of 128 SARS-CoV-2 RNA-positive corpses, 20% still retained infectious viruses up to 14 days postmortem (5). Collectively, these results demonstrate that infectious viruses remain in corpses. In this study, the wrapped carcass was placed on bedding in the cage in which the live naive hamsters were placed. Therefore, the naive animals could contact the wire net in which the ethanol-disinfected carcass was wrapped. However, because there was a gap between the wire net and the mouth and nose of the carcass, it was not possible for the contact animals to directly touch the mouth and nose of the carcass. Moreover, we did not observe emission of fluids postmortem. Within a few hours of death, a dead body begins to retain postmortem gases in the gastrointestinal tract (7). In this study, we confirmed that angel care, in which the pharynx, nostrils, and rectum are plugged, was effective in preventing the leakage of gas containing SARS-CoV-2 from a dead hamster in addition to preventing the leakage of bodily fluids. Therefore, it is possible that infectious viruses are transmitted via the postmortem gases produced during the decomposition process or by other postmortem changes in the dead body.

Rodic et al. reported that COVID-19 nucleic acids were identified from the lungs of embalmed corpses (8). However, viral RNA detection does not distinguish between viable and dead viruses (5, 9, 10). Persistently positive reverse transcriptase PCR (RT-PCR) does not indicate whether infectious virus is still present in a person’s body. SARS-CoV-2 RNA can be detected beyond the infectious period (5, 9). Therefore, detection of viral RNA does not necessarily indicate infectivity. It is known that formaldehyde and glutaraldehyde inactivate SARS-CoV-2 (11, 12). The embalming agent used in this study contained 7% formaldehyde and 4% glutaraldehyde. Therefore, most of the virus in the dead body was likely inactivated by these chemicals. In addition, embalming is a process that prevents decomposition and the formation of postmortem gases. Both functions may have prevented the transmission of SARS-CoV-2 from the dead bodies.

In this study, we found that SARS-CoV-2 could be transmitted from a dead body. Further studies are needed to determine the source of infection (e.g., postmortem gases). However, we also found that angel care or embalming could prevent transmission from a dead body. These results indicate that protection from infection is essential when handling infected corpses and that the appropriate treatment of SARS-CoV-2-infected corpses is important.

## MATERIALS AND METHODS

### Cells and virus.

VeroE6/TMPRSS2 (13) (JCRB 1819) cells were propagated in Dulbecco’s modified Eagle medium (DMEM) containing 10% fetal calf serum (FCS), 1 mg/mL Geneticin (G418; Invivogen), and 5 μg/mL Plasmocin prophylactic (InvivoGen). The VeroE6/TMPRSS2 cells were incubated at 37°C with 5% CO_2_ and regularly tested for mycoplasma contamination by PCR; they were confirmed to be mycoplasma free. SARS-CoV-2/UT-NCGM02/human/2020 (Wuhan strain) was propagated in the VeroE6/TMPRSS2 cells in virus production serum-free medium (VP-SFM; Thermo Fisher Scientific).

All experiments with SARS-CoV-2 were performed in enhanced biosafety level 3 (BSL3) containment laboratories at the University of Tokyo, which are approved for such use by the Ministry of Agriculture, Forestry, and Fisheries (Japan).

### Experimental infection.

Six-month-old male Syrian hamsters (Japan SLC Inc., Shizuoka, Japan) were used for this study. Under ketamine-xylazine anesthesia, hamsters were inoculated with 10^3^ PFU (in 100 μL) of SARS-CoV-2 via the intranasal route. At 24 or 48 h postinfection, the hamsters were euthanized with deep anesthesia and cervical dislocation. To disinfect viruses on the surface of the bodies, the entire bodies were immersed in an alcohol bath for 30 s. The bodies were then wrapped with wire net to prevent them from being cannibalized by cohousing hamsters (Fig. 1A). One wrapped body and two naive hamsters were cohoused as one group in the same cage. As a control, one live infected hamster and two naive hamsters were also cohoused (Fig. 1B). Twenty-four hours after cohousing, the wrapped body and the live infected hamster were removed from the cages, and the lungs and nasal turbinates were collected for virus titration. The remaining naive hamsters were euthanized 3 days after removal of the infected hamster, and their organs were collected for virus titration. The animal room was kept at 25°C and 50% humidity.

### Angel care.

The bodies were dipped in alcohol prior to receiving angel care. Three hundred microliters of jelly for human angel care (Humex Co., Ltd., Japan) was inserted into the mouth and plugged with cotton; the nostrils and rectum were plugged with medical-grade Aron Alpha adhesive.

### Embalming.

The bodies were dipped in alcohol prior to embalming. The heart was exposed, and embalming agents (The Dodge Company, USA) were injected through the apex of the heart. The inguinal vein was exposed, and blood was drained through it. The wound was sutured with a medical stapler.

### Plaque assay.

The lungs and nasal turbinates were homogenized in 1.0 mL of growth medium and clarified by centrifugation (1,000 × *g* for 5 min). Confluent monolayers of VeroE6/TMPRSS2 cells were infected with 100 μL of undiluted homogenates or 10-fold dilutions (10^−1^ to 10^−5^) of homogenates and incubated for 1 h at 37°C. After the inoculum was removed, the cells were washed with growth medium and overlaid with a 1:1 mixture of 2× growth medium and 2% agarose. The plates were incubated at 37°C for 48 h before virus plaques were counted.

### Ethics statements.

The research protocol for the animal studies was in accordance with the Regulations for Animal Care at the University of Tokyo (Tokyo, Japan) and was approved by the Animal Experiment Committee of the Institute of Medical Science, University of Tokyo (approval number PA20-30).
